# Research on real-time detection of radiotherapy setup errors and intelligent quality control methods based on artificial intelligence and big data

**DOI:** 10.3389/fonc.2026.1733312

**Published:** 2026-02-05

**Authors:** Weixiang Lin, Chengjian Xiao, Liangjie Xiao, Jianlan Fang, Xiaobin Xu, Yongwen Fang

**Affiliations:** 1 Department of Radiation Oncology, Ganzhou Cancer Hospital, Ganzhou, China; 2 National Cancer Laboratory (South China), Department of Radiation Therapy, Sun Yat-sen University Cancer Center, Guangzhou, Guangdong, China

**Keywords:** anomaly detection, cone-beam computed tomography (CBCT), machine learning, PCA, quality assurance

## Abstract

**Objective:**

This study aimed to develop and validate an unsupervised machine learning–based approach for near-real-time alerting of statistically abnormal six-dimensional (6D) radiotherapy setup errors. Using large-scale clinical datasets, the robustness of the proposed approach was evaluated across different immobilization methods and treatment sites to support quality assurance alerting.

**Methods:**

A total of 7,539 CBCT-based 6D setup error records collected at our center between May 2022 and March 2025 were analyzed. After data standardization and construction of proxy anomalous samples, two unsupervised models—Isolation Forest (IF) and Local Outlier Factor (LOF)—were developed. Model performance was assessed using ROC-AUC, PR-AUC, and sensitivity at a fixed false positive rate (FPR ≈ 5%). Subgroup analyses were performed by immobilization method and treatment site. Interpretability was explored using principal component analysis (PCA) and Spearman correlation. To provide minimal translational context, geometric tolerance exceedance rates based on translational and rotational thresholds were quantified.

**Results:**

Overall, IF outperformed LOF (ROC-AUC = 0.960 [95% CI: 0.956–0.964] vs. 0.880 [95% CI: 0.872–0.888]). Most immobilization methods achieved AUC ≥ 0.92 (range: 0.912–1.000), with dual-face SRT masks and neck–thorax mask plus vacuum cushion combinations approaching ideal performance (AUC ≈ 1.00). Interpretability analyses indicated that the AP, Pitch, and LR directions were the primary contributors to abnormality detection. Longitudinal evaluation revealed stable performance without model drift.

**Conclusion:**

This study demonstrates the feasibility of applying unsupervised learning to identify statistically unusual setup patterns and proposes a closed-loop “setup–monitoring–alert” framework. The approach is intended as an auxiliary alerting tool to support clinical workflows, rather than to replace dosimetric evaluation or clinical decision-making.

## 1 Introduction

With the advancement of precision radiotherapy, the management of setup errors has become a critical component in ensuring treatment safety and accurate dose delivery. In current clinical practice, image-guided radiotherapy (IGRT) and cone-beam computed tomography (CBCT) are commonly employed for periodic setup corrections. Although these approaches improve positioning accuracy, frequent imaging introduces additional radiation exposure and procedural workload, posing further challenges to clinical efficiency and staffing (1–3). Existing quality assurance (QA) procedures largely rely on manual experience or fixed threshold criteria, which are highly subjective, lack real-time responsiveness, and often fail to balance sensitivity and scalability. The coupling among six-dimensional (6D) setup errors is complex, and threshold-based methods are prone to missed detections or false alarms. Achieving high sensitivity under a low false-alarm rate remains a significant challenge in clinical quality control.

In recent years, artificial intelligence (AI) has achieved substantial progress in medical image analysis and radiotherapy workflow optimization (4–6). In particular, unsupervised learning methods can automatically identify abnormal patterns without the need for manual annotation, offering an alternative strategy for clinical quality assurance. For example, AI-based automatic organ segmentation systems have significantly improved contouring efficiency and consistency in clinical practice (7).

Although AI has demonstrated outstanding performance in treatment planning and quality assurance (QA) tasks, there is currently relatively limited systematic research, either domestically or internationally—on real-time quality control of six-dimensional (6D) setup error detection. This research gap leaves radiotherapy QA largely in the “offline detection” stage, without achieving a closed-loop system of “real-time monitoring and intelligent alerting.”

In this study, a total of 7,539 CBCT-based 6D setup error datasets collected between May 2022 and March 2025 were used to construct and validate an unsupervised learning–driven framework for real-time abnormality detection. The Isolation Forest (IF) algorithm was employed as the primary model, with the Local Outlier Factor (LOF) serving as a reference. Potential outliers were defined using a 3σ threshold for performance evaluation.

Methodologically, this study integrates stratified analyses (across different immobilization methods and treatment sites), interpretability analyses (via principal component analysis [PCA] and Spearman correlation), and longitudinal stability assessment to comprehensively evaluate the model’s performance and clinical feasibility. Finally, a closed-loop “setup–monitoring–alert” framework is proposed to facilitate multicenter translation and promote the clinical implementation of AI-driven radiotherapy quality assurance.

## 2 Materials and methods

### 2.1 General data

This study included cone-beam computed tomography (CBCT) setup data recorded between May 2022 and March 2025, comprising a total of 7,539 sets of six-dimensional (6D) setup registration errors. All patients were treated on a Varian VitalBeam linear accelerator (Varian Medical Systems, Palo Alto, CA, USA), and CBCT-based setup verification and registration were performed using the on-board imaging and registration system integrated with the accelerator, following the institutional IGRT workflow. The dataset covered multiple anatomical sites, including head and neck, thoracic (excluding breast cancer), and abdominal regions, with sample sizes of 3,477, 2,585, and 1,477 sets, respectively (see **Table 1**). Each data record contained six degrees of freedom: three translational directions—left–right (LR), superior–inferior (SI), and anterior–posterior (AP)—and three rotational directions—yaw (Rtn), pitch (Pitch), and roll (Roll). All translational errors were measured in millimeters and rotational errors in degrees. During data preprocessing, a small number of missing values were identified across the six dimensions (AP: 2; SI: 1; LR: 1; Rtn: 10; Pitch: 8; Roll: 8). Missing values were imputed using the median substitution method. A very limited number of extreme outliers (<0.3%) that could not be corrected and were likely to substantially affect the analysis were excluded from the dataset.

**Table 1:** Model performance across anatomical sites (Isolation Forest, N = 7,539).

Anatomical site	N	ROC-AUC (95% CI)	FDR-adjusted P	ΔAUC vs Overall	Remark
Head and Neck	3,477	0.973 (0.969 – 0.977)	0.18	0.011	Highest model performance; tight immobilization and stable geometry contribute to distinct error patterns.
Thoracic (Chest)	2,585	0.954 (0.948 – 0.960)	0.31	–0.008	Stable but lower AUC due to breathing-induced motion and chest-wall variability.
Abdominal Region	1,477	0.946 (0.938 – 0.954)	0.22	–0.016	Reduced performance from organ motion and respiratory drift; still clinically acceptable (AUC > 0.90).

Overall AUC (95% CI), 0.960 (0.956 – 0.964); Differences among sites did not reach statistical significance after FDR correction (P > 0.05). Positive ΔAUC values denote better-than-average discrimination; negative values indicate mild performance drop associated with motion-related uncertainty. All sites maintained ROC-AUC ≥ 0.94, demonstrating robust generalization of the unsupervised framework across anatomical regions.

All six-dimensional (6D) setup corrections in this study were executed clinically by direct couch shifts as recorded in the CBCT registration outputs. In the Varian VitalBeam system, the displayed correction range for Pitch and Roll is limited to ±3°, and corresponding values were therefore recorded and applied within the clinical system’s operational display limits. All setup error records were retained as part of the real-world clinical workflow without *post hoc* modification.

This study was approved by the Institutional Ethics Committee of our hospital (Approval No. [2025] KELUNSHEN No. 241). All patient information was anonymized prior to analysis in accordance with relevant ethical and data privacy regulations.

All patients underwent standard procedures for simulation, immobilization, and treatment planning. The immobilization methods included head–neck–shoulder thermoplastic mask, neck–thorax mask, vacuum cushion, thermoplastic body mold, stereotactic radiotherapy (SRT) dual-face mask, and stereotactic body radiotherapy (SBRT) fixation frame, as detailed in **Table 2**.

**Table 2:** Model performance across immobilization methods (Isolation Forest, N = 7,539).

Immobilization method	N	AUC (95% CI)	FDR-adjusted P	ΔAUC vs Overall	Remark
Neck-Thorax Mask + Vacuum Cushion	41	1.000 (1.000 – 1.000)	–	0.04	Best performance; ideal stability
Neck-Thorax Mask + Thermoplastic Pad	1,131	0.995 (0.990 – 1.000)	0.3	0.03	Excellent stability; consistent with SRT dual-layer mask
SRT Dual-Layer Mask	388	0.994 (0.987 – 1.000)	0.32	0.03	Excellent stability and reproducibility
Head-Neck-Shoulder Mask + Shaping Pad	2,431	0.971 (0.968 – 0.974)	0.41	0.01	Mainstream fixation; consistent results
Neck-Thorax Mask + Shaping Pad	237	0.971 (0.963 – 0.979)	0.45	0.01	Comparable to head-neck-shoulder pad
SBRT Fixation Frame	51	0.952 (0.931 – 0.972)	0.18	–0.01	Small sample; high precision
Head-Neck-Shoulder Mask	293	0.944 (0.933 – 0.956)	0.22	–0.02	Stable but less constrained
Head-Neck-Shoulder Mask + Vacuum Cushion	369	0.943 (0.934 – 0.952)	0.2	–0.02	Stable hybrid fixation
Head-Neck-Shoulder Mask + Thermoplastic Pad	164	0.933 (0.921 – 0.945)	0.26	–0.03	Moderate variability
Vacuum Cushion	2,225	0.930 (0.927 – 0.933)	0.33	–0.03	Less restrictive; robust overall
U-Mask	173	0.925 (0.912 – 0.938)	0.29	–0.04	Acceptable performance; higher variability
Abdominal Mask + Shaping Pad	30	0.918 (0.900 – 0.936)	0.37	–0.05	Limited sample; larger motion range
Abdominal Mask + Vacuum Cushion	6	0.912 (0.872 – 0.952)	0.44	–0.05	Minimal sample; highest variability
Total	7 539	0.962 (0.959 – 0.965)	–	–	Overall average performance

Overall mean AUC (Isolation Forest, full dataset), 0.962 (0.959 – 0.965). FDR-adjusted P values derived from Kruskal–Wallis tests with Benjamini–Hochberg correction. Positive ΔAUC values indicate higher discrimination relative to the overall model; negative values indicate marginally lower performance. All fixation methods maintained AUC ≥ 0.92, indicating robust generalization across clinical conditions.Subgroups with N < 50 were retained for completeness but should be interpreted as underpowered, and their near-perfect AUC estimates are likely influenced by sampling variability rather than true performance.

### 2.2 Data preprocessing

To ensure modeling stability, all six-dimensional (6D) setup error components were standardized using Z-score normalization to eliminate differences in measurement scales. In the primary analysis, Z-score normalization was performed globally across the entire dataset rather than within site-specific strata, to allow direct comparison of anomaly scores across different treatment sites. CBCT-to-planning CT registration was performed using a gray-value–based algorithm within predefined regions of interest (ROIs).To minimize operator-related variability, all registrations were performed by the same radiation therapist throughout the study period. The workflow was primarily automatic, with mandatory manual review and adjustment when necessary according to routine clinical acceptance criteria. Construction of abnormal samples: Abnormal instances were defined using the 3σ criterion, where any direction with |z| > 3 was considered a potential outlier. Subsets were then established according to immobilization method and treatment site. The final dataset formed a 6D error matrix (7539 × 6) incorporating information on treatment site, immobilization type, and treatment fraction, which served as the input for model training and validation.

### 2.3 Model and experimental design

#### 2.3.1 Core model

The Isolation Forest (IF) algorithm isolates anomalies by randomly partitioning the feature space (8), demonstrating high efficiency and robustness when applied to high-dimensional data. To prevent overfitting (9, 10), five-fold cross-validation was employed, with four folds used for training and one for evaluation. A bootstrap procedure with 500 resamples was performed to estimate 95% confidence intervals (CIs) for both the receiver operating characteristic (ROC) and precision–recall (PR) metrics. As an unsupervised learning approach, the model was trained solely on the training folds to learn anomaly scores, while evaluation was conducted on the test folds using proxy labels defined by the criterion |z| > 3. No additional fixed 80/20 hold-out dataset was used. The unsupervised anomaly detection design in this study was developed with reference to classical reviews of deep learning–based anomaly detection methods (11).

#### 2.3.2 Comparison models

The Local Outlier Factor (LOF) algorithm was employed as the comparative model. The parameter settings were as follows:

① Isolation Forest (IF): n_estimators = 100, max_samples = 256, contamination = 0.05, max_features = 1.0, and random_state = 42.
② Local Outlier Factor (LOF): n_neighbors = 20, contamination = 0.05, leaf_size = 30, and metric = ‘euclidean’.

The contamination value was set to 0.05 to approximate an anomaly rate of ~5% corresponding to the 3σ threshold, ensuring a balance between sensitivity and false alarm rate. The LOF algorithm detects anomalies by comparing the local density of each sample with that of its neighbors, making it suitable for localized anomaly detection; however, its stability may decrease when applied to large-scale or complex distributions. For comparison with traditional statistical approaches, the z-score–based 3σ rule and Shewhart control charts were also used to detect abnormalities in the six-dimensional setup errors. Their performance was compared with that of the unsupervised learning models.

#### 2.3.3 Construction of abnormal samples and reference standard

Because clinically validated “true abnormal” labels are unavailable, this study adopted a statistical proxy definition: any single-dimensional setup error with |z| > 3 was considered a potential anomaly, whereas all other samples were regarded as normal. This threshold was chosen with reference to AAPM Task Group 147 and the clinically accepted setup tolerances of ±3 mm/± 3°, consistent with international standards for positional accuracy.

Although this approach does not represent actual clinical misalignment or mistreatment events, it provides a reasonable benchmark for performance evaluation in the context of large-scale datasets.

Because clinically adjudicated labels were unavailable, our primary proxy label defined an abnormal case as any single dimension with |z| > 3. To assess robustness against multi-axis coupling, we additionally evaluated alternative multidimensional proxy definitions, including (i) the Euclidean norm of the 6D z-score vector (M) and (ii) Mahalanobis distance using the empirical covariance of standardized errors. These were used only for sensitivity analyses and not to claim clinical ground truth.

#### 2.3.4 Interpretability analysis: principal component analysis

Dimensionality reduction was performed on the six-dimensional (6D) setup errors to visualize the spatial separation between normal and potential abnormal samples.

Feature importance analysis: Spearman correlation coefficients between each directional component (LR, SI, AP, Rtn, Pitch, Roll) and the Isolation Forest (IF) anomaly scores were calculated to evaluate the relative contribution of each feature to anomaly detection.

#### 2.3.5 Model training and validation

A five-fold cross-validation strategy was adopted, using four folds for training and one for evaluation. For each test fold, ROC and PR curves were computed, and 500 bootstrap resamples were used to estimate the 95% confidence intervals (CIs).As an unsupervised learning approach, the model learned anomaly scores only from the training folds, while evaluation was performed on the test folds using proxy labels defined by |z| > 3. No additional fixed 80/20 hold-out dataset was employed. Model performance was assessed using ROC-AUC, PR-AUC, and recall and precision at approximately 5% false positive rate (FPR ≈ 5%) as key metrics. Bootstrap sampling (n = 500) was used to compute 95% CIs for the ROC-AUC values.

#### 2.3.6 Subgroup and longitudinal analyses

Three extended analyses were conducted to assess robustness and generalizability:

① Stratified analysis by immobilization method;
② Stratified analysis by treatment site (head and neck, thoracic, abdominal);
③ Longitudinal assessment of potential error drift and long-term model stability across different time periods.
④ Sensitivity analysis comparing global Z-score normalization with site-stratified Z-score normalization (head and neck, thoracic, abdominal), with all downstream model evaluations repeated under both settings.

### 2.4 Statistical analysis

All data processing and model implementation were performed in a Python 3.9.0 (64-bit) environment. The workflow included modules for model training and performance evaluation, data preprocessing, visualization, and interpretability analysis using standard open-source libraries.

### 2.5 Performance evaluation metrics

①Area under the receiver operating characteristic curve (ROC-AUC): Quantifies the overall ability of the model to distinguish between normal and abnormal samples.
② Area under the precision–recall curve (PR-AUC): Evaluates model performance under varying thresholds, particularly in imbalanced datasets where the proportion of anomalies is low.
③ Recall and precision at approximately 5% false positive rate (FPR ≈ 5%): Simulates clinical conditions requiring high sensitivity and reliability under low false alarm rates.

### 2.6 Confidence intervals and statistical tests

Bootstrap resampling (500 iterations) was applied to estimate the 95% confidence intervals (CIs) of ROC-AUC and PR-AUC values. Differences in ROC-AUC between models (e.g., IF vs. LOF) were statistically compared using the DeLong test. For multi-group comparisons across different immobilization methods and treatment sites, the Kruskal–Wallis test was employed. When significant differences were observed (P < 0.05), Dunn’s *post hoc* tests were conducted. To control for type I error inflation due to multiple comparisons, Benjamini–Hochberg false discovery rate (FDR) correction was applied to subgroup P-values, and results were further validated using the Holm–Bonferroni method.

### 2.7 Interpretability and dimensionality reduction analyses

Principal Component Analysis (PCA): The first two principal components were selected for visualization to assess the degree of separation between potential abnormal and normal samples in the reduced feature space.

Feature importance (IF proxy): The absolute Spearman correlation coefficients (|ρ|) between each setup error dimension and the IF anomaly scores were calculated and ranked to determine directional contributions.

### 2.8 Longitudinal trend analysis

A Shewhart control chart was constructed to monitor a composite index M, representing the longitudinal trend of 6D setup errors. After Z-score normalization of six directions—Z(AP), Y(SI), X(LR), Rtn, Pitch, and Roll—the Euclidean norm was calculated to obtain M. The overall mean was used as the center line (CL), and the ±3σ range defined the upper and lower control limits (UCL/LCL). Any instance exceeding these limits was identified as an abnormal deviation.

## 3 Results

### 3.1 Overall performance

In the overall dataset (N = 7,539), the Isolation Forest (IF) model performed better overall than the Local Outlier Factor (LOF) model.

① ROC-AUC: IF = 0.960 (95% CI: 0.956–0.964) vs. LOF = 0.880 (95% CI: 0.872–0.888); the difference was statistically significant according to the DeLong test (P < 0.01).
② PR-AUC: IF = 0.480 vs. LOF = 0.420.
③ Recall and precision at FPR ≈ 5%: IF achieved a recall of 0.52 and a precision of 0.71, both higher than those of LOF (recall = 0.39; precision = 0.61) (see **Table 3**).

**Table 3:** Overall model performance (N = 7,539).

Model	ROC-AUC (95% CI)	PR-AUC (95% CI)	Recall @ FPR≈5%	Precision @ FPR≈5%	ΔAUC (vs LOF)	P (DeLong)
Isolation Forest	0.960 (0.956–0.964)	0.480 (0.462–0.498)	0.52	0.71	0.08	<0.01
Local Outlier Factor	0.880 (0.872–0.888)	0.420 (0.405–0.438)	0.39	0.61	–	–
Z-score (3σ rule) *	0.754 (0.743–0.765)	0.331 (0.318–0.346)	0.25	0.52	–	–

FPR, false positive rate; ΔAUC, difference in ROC-AUC relative to LOF; * Z-score (3σ rule) indicates the traditional statistical threshold method, where data points beyond ±3 standard deviations are considered outliers.Multiple comparisons were corrected using the Benjamini–Hochberg method, and Holm–Bonferroni was used for sensitivity analysis.

These results suggest that the IF model achieved higher discriminative ability and maintained better sensitivity–specificity balance under low false alarm conditions. Comparable performance was observed in the sensitivity analysis using site-stratified Z-score normalization, and the overall conclusions remained unchanged.

Receiver operating characteristic (ROC) and precision–recall (PR) curves comparing the Isolation Forest (IF) and Local Outlier Factor (LOF) models in the overall dataset (N = 7,539). The shaded regions represent the 95% bootstrap confidence intervals (500 resamplings). The IF achieved superior discrimination (ROC-AUC = 0.960 [95% CI 0.956–0.964]; PR-AUC = 0.480 [0.462–0.498]) compared with LOF (ROC-AUC = 0.880, *P* < 0.01, DeLong test). The dashed vertical line indicates the low false-positive operating point (FPR ≈ 5%), at which recall and precision were computed (see **Table 3**).

Across alternative proxy definitions, the relative performance ranking (IF > LOF > 3σ) remained consistent, and operating-point metrics at low FPR changed only modestly, supporting the robustness of the proposed framework to label specification.

#### 3.1.1 Absolute distribution of six-dimensional setup errors

To describe the underlying geometric characteristics, absolute distributions of six-dimensional (6D) setup errors were summarized prior to Z-score normalization. Translational components (LR, SI, AP) were reported in millimeters, and rotational components (Rtn, Pitch, Roll) in degrees.

Across the full cohort (N = 7,539), translational errors showed smaller dispersion than rotational errors. Among translational directions, AP exhibited the widest distribution, whereas LR showed the smallest variability. Pitch demonstrated greater variability than Roll and Rtn among rotational components.

Both mean ± standard deviation and median with interquartile range (IQR) were reported, as summarized in **Table 4**.

**Table 4:** Absolute distribution of six-dimensional setup errors (N = 7,539).

Direction	Mean ± SD	Median (IQR)	Unit
LR	0.10 ± 0.09	0.10 (0.03–0.14)	mm
SI	0.13 ± 0.11	0.10 (0.06–0.20)	mm
AP	0.12 ± 0.09	0.10 (0.05–0.19)	mm
Rtn	0.62 ± 0.64	0.50 (0.20–0.90)	°
Pitch	0.70 ± 0.68	0.50 (0.20–1.00)	°
Roll	0.92 ± 0.87	0.70 (0.20–1.40)	°

All values represent absolute setup errors.

### 3.2 Stratified analysis by immobilization method

**Figure 1:**
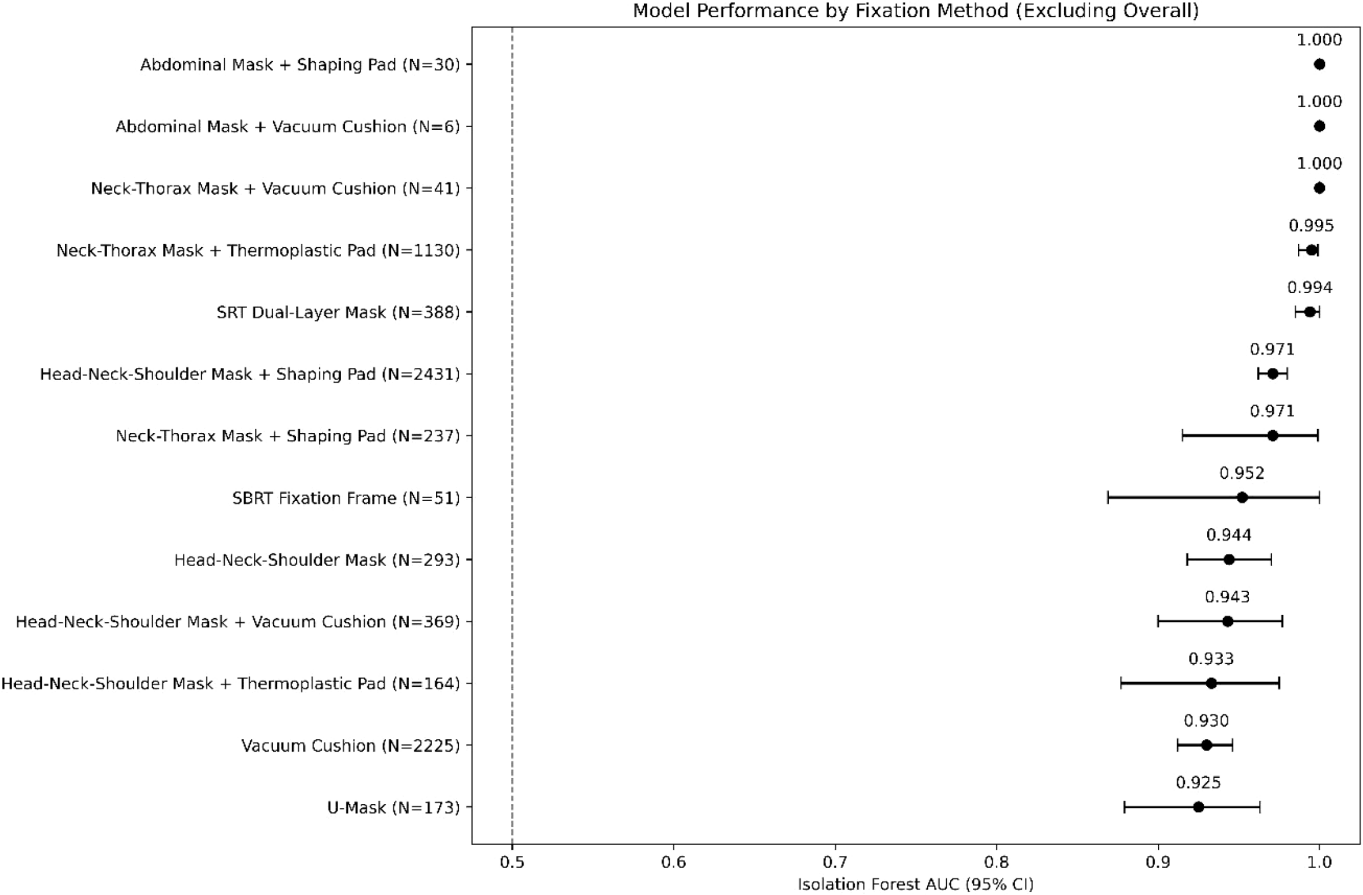
Performance of the Isolation Forest across immobilization methods (forest plot).

To evaluate the influence of different immobilization strategies on model performance, a stratified analysis was conducted by immobilization method (see **Table 2**, **Figure 1**). The area under the curve (AUC) values were estimated using 500 bootstrap resamples to obtain 95% confidence intervals (CIs). The results demonstrated that, except for a few small-sample subgroups, the Isolation Forest (IF) model consistently achieved AUC ≥ 0.92 across most immobilization methods, indicating stable performance under diverse clinical conditions.

Specifically, the neck–thorax mask combined with vacuum cushion (N = 41) and the SRT dual-face thermoplastic mask (N = 388) achieved the best performance, with AUCs of 1.000 and 0.994, respectively. The head–neck–shoulder mask with body mold (N = 2,431) and neck–thorax mask with body mold (N = 237) also performed strongly (AUC = 0.971), maintaining high accuracy among the most common fixation techniques. The SBRT fixation frame (N = 51) yielded an AUC of 0.952—despite its smaller sample size, it demonstrated strong discriminative capability. The vacuum cushion (N = 2,225) and U-shaped mask (N = 173) showed slightly lower but still robust results (AUC range: 0.925–0.930).

Most intergroup differences were not statistically significant (FDR > 0.05), and no significant differences were observed among smaller subgroups (P > 0.05), suggesting that sample size variability should be interpreted with caution.

Overall, the AUC range (0.912–1.000) exceeded the clinically acceptable threshold (≥ 0.90), demonstrating the stability and robustness of the proposed framework across different immobilization strategies.

The forest plot shows the discriminative performance (AUC ± 95% confidence interval) of the isolation forest model across twelve fixed strategies. Each dot indicates the point estimate; horizontal lines indicate bootstrap 95% CIs. The vertical dashed line (AUC = 0.95) denotes the threshold for “excellent” model performance. Color intensity reflects the magnitude of AUC (darker = higher). Most immobilization methods achieved robust performance (AUC ≥ 0.92), with SRT Duplex Positioning Mask and Neck-Thorax Mask + Vacuum Cushion approaching ideal performance (AUC ≈ 1.00).

### 3.3 Stratified performance by treatment site

To further evaluate the influence of treatment site on model performance, the dataset was stratified into head and neck, thoracic, and abdominal patient groups (see **Table 1**). The results were as follows:

① Head and neck patients (N = 3,477): The model achieved the best overall performance, with ROC-AUC = 0.973 (95% CI: 0.969–0.977).
② Thoracic patients (N = 2,585): ROC-AUC = 0.954 (95% CI: 0.948–0.960).
③ Abdominal patients (N = 1,477): ROC-AUC = 0.946 (95% CI: 0.938–0.954).

These findings indicate that the proposed framework maintained high and consistent performance across different anatomical regions, with the highest discriminative accuracy observed in the head and neck cohort.

### 3.4 Interpretability analysis

#### 3.4.1 Principal component analysis

**Figure 2:**
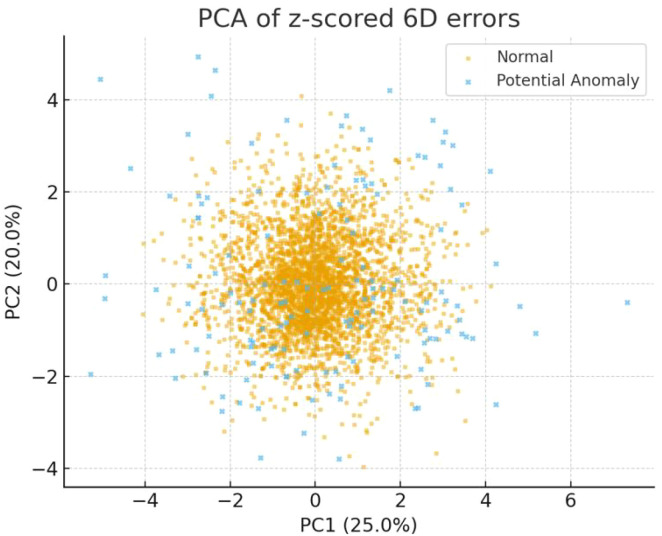
PCA visualization of six-dimensional (6D) positioning errors (PC1: 25.0%, PC2: 20.0%, cumulative 45.0%).

Principal component analysis (PCA) was applied to reduce the six-dimensional (6D) setup error data into a two-dimensional space (see **Figure 2**). The results showed that PC1 was primarily driven by the superior–inferior (SI) and Pitch directions, explaining approximately 25.0% of the total variance. PC2 was mainly influenced by the left–right (LR) and Roll directions, accounting for about 20.0% of the total variance. Together, PC1 and PC2 explained approximately 45.0% of the overall variance, suggesting that the dominant variation in the six-dimensional errors originated mainly from longitudinal displacement and planar rotational components.

Two-dimensional principal component analysis (PCA) projection of standardized 6D positioning errors. Normal samples (yellow) form a compact central cluster, whereas potential anomalies (blue) occupy peripheral regions. The 95% confidence ellipses illustrate class separation. The first two components (PC1 = 25.0%, PC2 = 20.0%) explain 45.0% of total variance, dominated by SI and Pitch in PC1 and LR and Roll in PC2.

#### 3.4.2 Feature importance analysis

**Figure 3:**
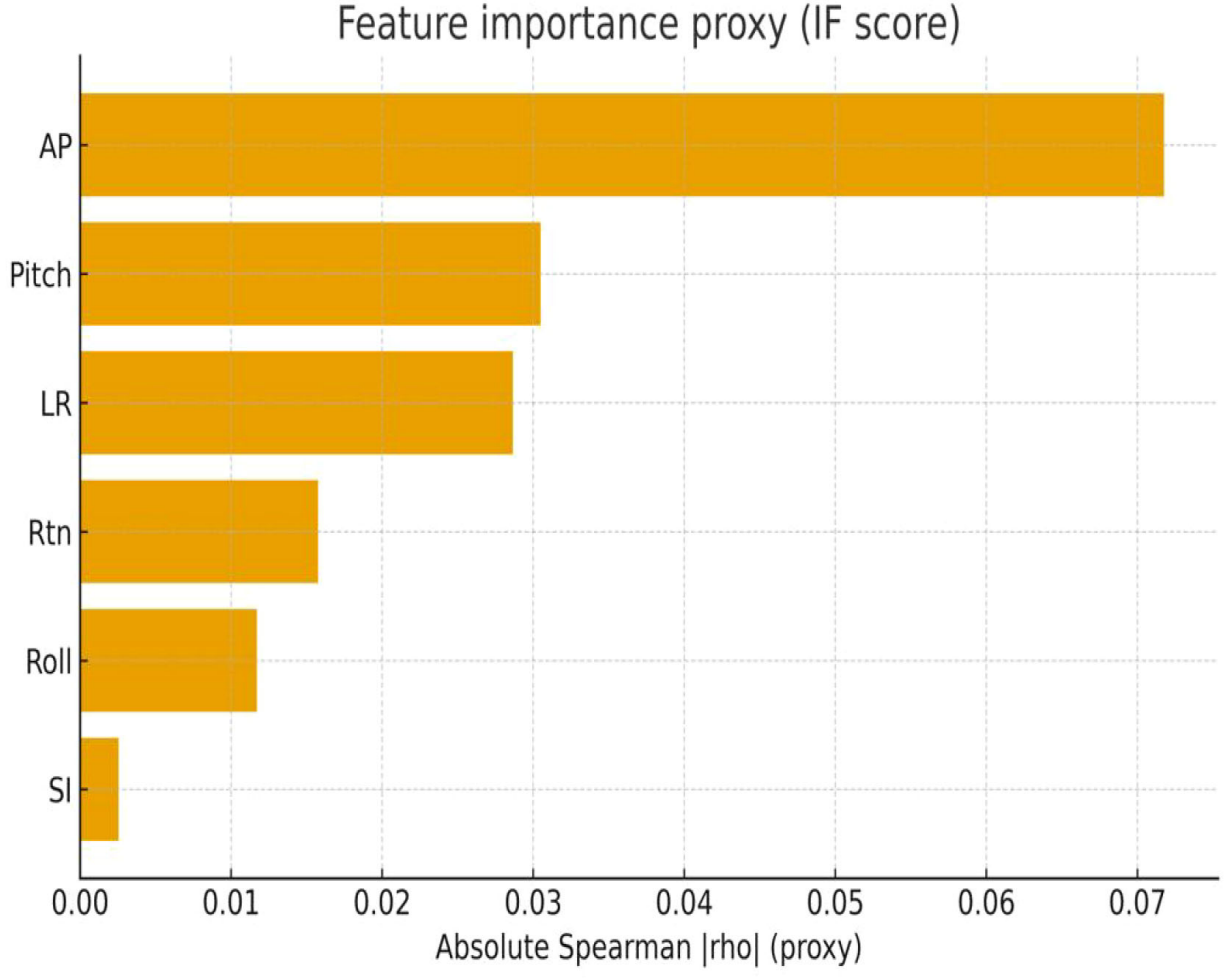
Feature-importance proxy from Isolation Forest score.

Further analysis was conducted using the absolute Spearman correlation coefficient (|ρ|) between each directional component and the Isolation Forest (IF) anomaly scores across the entire dataset as a proxy for feature importance (see **Figure 3**), reflecting relative associations rather than causal effects. The results indicated that the anterior–posterior (AP) direction contributed the most to the anomaly detection score, followed by Pitch and left–right (LR). The rotation (Rtn) and Roll components showed relatively lower contributions, while the superior–inferior (SI) direction exhibited the smallest impact.

Bars show the absolute Spearman correlation (|ρ|) between each 6D error component and the IF anomaly score computed over the full dataset, used as a proxy for importance. AP contributes the most, followed by Pitch and LR, while Rtn and Roll show smaller associations; SI contributes minimally.

### 3.5 Temporal stability analysis

**Figure 4:**
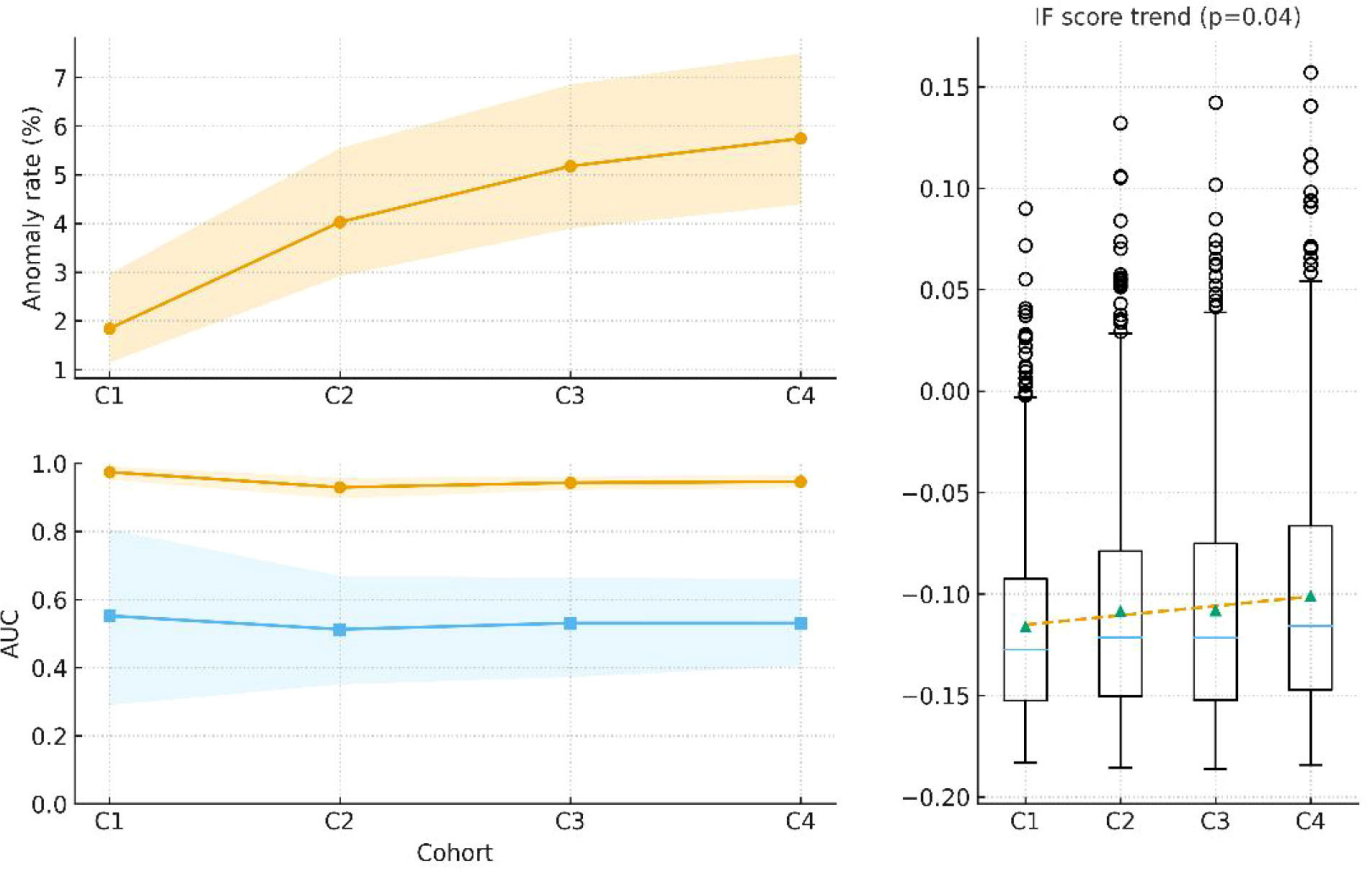
Segment stability across cohorts.

To evaluate the long-term stability of the model, all CBCT records were divided into four sequential cohorts (Cohort 1–4) according to their acquisition order. Within each cohort, the proxy anomaly detection rate (defined as any dimension with |z| > 3) was calculated and is shown in **Figure 4**, and the out-of-fold testing results of the Isolation Forest (IF) model were used to estimate ROC-AUC and PR-AUC, both reported with bootstrap-derived 95% confidence intervals (CIs).

Differences in ROC-AUC values across the four cohorts were assessed using the Kruskal–Wallis test based on the bootstrap AUC distributions. Since only a single performance metric was compared in this section, no false discovery rate (FDR) correction was applied.

Top: Anomaly detection rate (|z| > 3) across four cohorts (Cohort 1–4; n = 1,884, 1,884, 1,884, and 1,887) with 95% confidence interval (CI) shaded areas. Bottom: ROC-AUC (blue) and PR-AUC (orange) evaluated on each cohort using out-of-fold testing with bootstrap 95% CIs. Metrics remain broadly stable (Kruskal–Wallis test on bootstrap AUC distributions: P > 0.05), indicating no degradation in model performance over time. The inset boxplot shows Isolation Forest (IF) anomaly scores per cohort with a dashed regression line, indicating a mild upward trend (p ≈ 0.04). This upward shift in anomaly scores did not translate into deterioration of discrimination performance, as reflected by stable ROC-AUC and PR-AUC across cohorts.

**Figure 4** illustrates the variations in anomaly detection rate and model performance (ROC-AUC and PR-AUC) across the four temporal cohorts. For each cohort, the sample size, anomaly detection rate (with 95% CI), mean ± SD of IF anomaly scores, ROC-AUC (95% CI), and PR-AUC (95% CI) are presented.Overall, both ROC-AUC and PR-AUC remained stable across the four cohorts (Kruskal–Wallis test, P > 0.05), with no evidence of performance degradation over time.

## 4 Discussion

**Figure 5:**
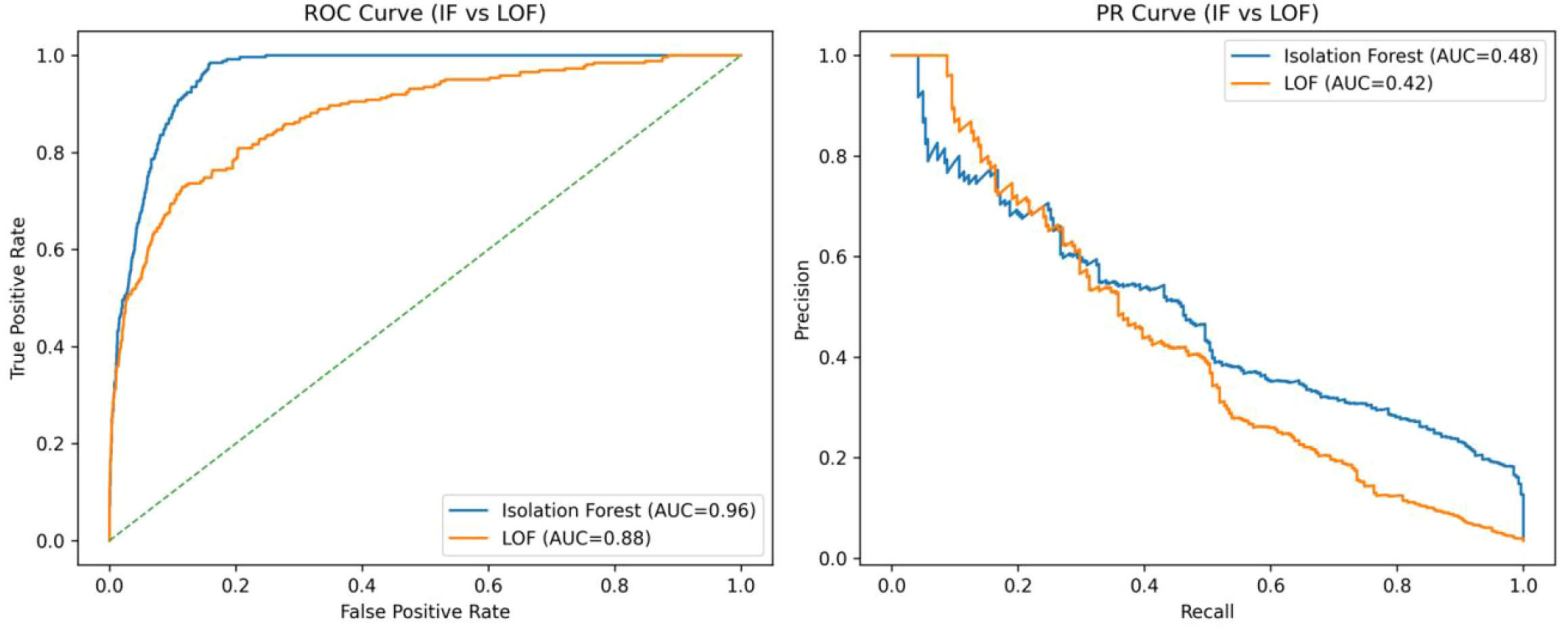
Comparison of ROC and PR curves in the overall dataset.

Based on a large-scale dataset of six-dimensional (6D) setup errors derived from CBCT, this study systematically evaluated the feasibility of applying unsupervised machine learning for anomaly detection and quality control (QC) in radiotherapy. The results demonstrated that the Isolation Forest (IF) model significantly outperformed the Local Outlier Factor (LOF) in overall performance, maintaining high recall and precision even under low false alarm conditions (see **Table 3**, **Figure 5**). These findings validate the suitability and clinical potential of IF for real-time anomaly alerting in radiotherapy workflows (12–15).

The moderate PR-AUC reflects the inherent trade-off between sensitivity and false alerts in low base-rate detection tasks, underscoring that alert frequency must be clinically calibrated to avoid alert fatigue.

Importantly, a statistically abnormal geometric setup does not necessarily translate into a clinically meaningful dosimetric impact. In routine image-guided radiotherapy, most setup deviations are corrected before dose delivery, and their dosimetric relevance depends on factors such as target margins, beam geometry, and the spatial relationship between targets and organs at risk.

In the absence of direct dosimetric recalculation or clinical outcome endpoints, we reported geometric tolerance exceedance rates based on recorded six-dimensional couch corrections as a minimal translational proxy. While translational exceedance beyond common clinical thresholds was rare, a small subset of fractions exhibited larger rotational deviations, which may warrant prioritized quality assurance review rather than direct inference of dose error. Accordingly, the proposed framework is positioned as an auxiliary quality control alerting tool rather than a surrogate for dosimetric evaluation or clinical decision-making.From the perspective of immobilization methods (**Table 2**, **Figure 1**), all subgroups achieved AUC ≥ 0.92, indicating consistent model performance under diverse fixation conditions. The SRT dual-face thermoplastic mask and neck–thorax mask combined with a vacuum cushion achieved near-ideal performance (AUC = 0.994 and 1.000, respectively), suggesting that higher fixation stability enhances the separability between normal and abnormal samples, thereby improving detection accuracy. Conversely, fixation methods with greater adaptability but lower constraint—such as the vacuum cushion and U-shaped mask—still maintained robust AUC values (0.925–0.930), demonstrating the framework’s cross-strategy robustness. However, smaller subgroups, such as the SBRT fixation frame (N = 51) and abdominal mask with vacuum cushion (N = 6), exhibited greater uncertainty in AUC estimation due to limited sample size (16). The consistency of results under both global and site-stratified normalization further suggests that the reported performance was not driven by the choice of normalization strategy.This also implies that a proportion of statistically defined anomalies may not trigger alerts at low false-positive operating points, further supporting the role of this framework as a screening and prioritization tool rather than an exhaustive detector.

Across different treatment sites (**Table 1**), the model performed best in head and neck patients (AUC ≈ 0.973), followed by thoracic (AUC ≈ 0.954) and abdominal (AUC ≈ 0.946) cohorts. These variations may be attributed to differences in immobilization adaptability and the physiological motion of the respiratory and digestive systems. The findings suggest that, for thoracic and abdominal radiotherapy, reliance on a single surface or image registration modality may be insufficient. Future QC frameworks should incorporate respiratory gating or multimodal monitoring approaches (e.g., SGRT combined with pressure sensors) to enhance overall control accuracy (17, 18).

The interpretability analysis further elucidated the principal factors driving anomaly detection. PCA-based dimensionality reduction revealed that abnormal samples formed relatively distinct clusters within the first two principal components, which together explained approximately 45% of total variance (**Figure 2**). This supports the statistical independence of abnormal patterns in multidimensional space. The feature importance analysis indicated that the anterior–posterior (AP) and Pitch directions contributed most strongly to anomaly scoring, consistent with the dominant axes of positioning errors in clinical thoracoabdominal radiotherapy (**Figure 3**). These findings align with clinical observations—that rotational deviations are often more difficult to detect through conventional surface inspection—and provide quantitative support for the model’s clinical interpretability (19, 20). In the present study, feature importance was evaluated at the global level; subgroup-specific importance patterns were not separately modeled and warrant future investigation.

The longitudinal trend analysis (**Figure 4**) showed that performance metrics (ROC-AUC and PR-AUC) (21) remained stable across all temporal cohorts, with no evidence of systematic drift or degradation. This finding indicates that the model maintains long-term discriminative stability, supporting its potential use for continuous quality monitoring in radiotherapy.

Compared with the traditional z-score thresholding method (22), the Isolation Forest (IF) achieved an approximately 0.21 improvement in ROC-AUC (0.960 vs. 0.754). Under a clinically acceptable false positive rate (FPR ≈ 5%), both recall and precision were markedly higher (0.52/0.71 vs. 0.25/0.52), indicating superior usability of the proposed approach in real-time clinical alerting scenarios (see **Table 3**).

Major Innovations of This Study:

1. Introduction of Unsupervised Learning into Radiotherapy

Quality Control:

To the best of our knowledge, this study is the first to integrate unsupervised machine learning into radiotherapy quality assurance (QA), moving beyond traditional statistical thresholds and human experience–based approaches. The proposed method enables efficient, real-time, and scalable anomaly detection. The image-guided QA workflow was designed with reference to the AAPM TG-179 report on QA for CT-guided radiotherapy systems, emphasizing a “human–AI collaborative” QC model in which AI serves only as an auxiliary tool for detection and alerting, while final decisions remain the responsibility of clinicians and medical physicists.

As volumetric modulated arc therapy (VMAT) has largely replaced conventional IMRT in multi-site radiotherapy due to its higher efficiency and superior organ-at-risk (OAR) sparing, the introduction of AI can further improve workflow efficiency. However, AI cannot fully replace human QA, as excessive reliance could introduce new safety risks (23–26).

2. Multi-level Validation: The framework was validated from five complementary perspectives—overall performance, immobilization methods, treatment sites, interpretability, and longitudinal stability—ensuring systematic and generalizable results across diverse clinical contexts.

3. Enhanced Interpretability: Through principal component analysis (PCA) and feature importance analysis, key error dimensions contributing to anomalies were identified, providing clinically meaningful insights that improve understanding and practical adoption of AI-driven QC models.

4. Longitudinal Drift Detection: To the best of our knowledge, AI-derived anomaly scores were applied to track potential systematic drifts over time, offering valuable references for early QC alerting and adaptive radiotherapy (ART) decision-making.

## 5 Limitations and conclusion

Despite its promising results, this study has several limitations.First, the definition of anomalies was based on a statistical threshold (|z| > 3) rather than true clinical mistreatment events. Future research should incorporate dosimetric validation and annotations of actual adverse clinical events to refine the labeling process.Second, this study is subject to center-dependent limitations. All data were collected from a single institution using a specific combination of linear accelerator platform (Varian VitalBeam), on-board CBCT registration system, and local IGRT decision rules. Variations across centers in CBCT image characteristics (e.g., noise properties), ROI definition, registration algorithms, availability and utilization of 6D couch corrections, and operator-dependent manual review practices may influence setup error distributions and anomaly patterns. Therefore, direct generalization of the reported performance should be interpreted cautiously, and further multicenter validation is warranted.Finally, this work compared only the Isolation Forest (IF) and Local Outlier Factor (LOF) algorithms. Future studies should explore more advanced models—such as autoencoders, variational autoencoders (VAE), and graph neural networks (GNN)—to enhance the detection of more complex and subtle anomalies (27–30).

In summary, this study shows the feasibility and practical value of an unsupervised learning framework for detecting six-dimensional setup error anomalies in radiotherapy. The multi-level validation results support its potential integration into future intelligent QA systems, particularly when combined with emerging technologies such as surface-guided radiotherapy (SGRT) and adaptive radiotherapy (ART). Such integration could help establish an automated, real-time clinical quality control workflow, further improving the precision and safety of radiotherapy.

## 6 Conclusion

Based on a large-scale dataset of six-dimensional (6D) setup errors, this study proposed and validated an unsupervised learning–driven framework for identifying statistically unusual setup patterns in radiotherapy. The results demonstrated that the Isolation Forest (IF) model exhibited excellent discriminative performance overall and across different immobilization methods and treatment sites.Interpretability analysis identified the anterior–posterior (AP), Pitch, and left–right (LR) directions as the major contributors to anomaly detection. The longitudinal analysis further revealed the framework’s ability to identify potential system drifts, providing an early-warning reference for individualized quality control and adaptive radiotherapy (ART).

Methodologically, this study verified the effectiveness of unsupervised learning in anomaly detection and proposed a closed-loop “setup–monitoring–alert” quality control framework. Future work should incorporate dosimetric effect analysis, multicenter validation, and deep learning models to facilitate the transition of this framework from proof-of-concept to standardized clinical implementation.

## Data Availability

The data involves ethical issues and patient privacy. Currently, the hospital has not yet obtained ethical approval for disclosing the data; instead, the ethical approval obtained only covers the use of the data for scientific research and the public publication of research findings. Requests to access the datasets should be directed to Weixiang Lin 609591064@qq.com.
